# Effects of Primary Kidney Disease Etiology on Renal Osteodystrophy in Pediatric Dialysis Patients

**DOI:** 10.1002/jbm4.10601

**Published:** 2022-04-08

**Authors:** Ornatcha Sirimongkolchaiyakul, Katherine Wesseling‐Perry, Barbara Gales, Daniela Markovic, David Elashoff, Georgina Ramos, Renata C Pereira, Mark R Hanudel, Isidro B Salusky

**Affiliations:** ^1^ Department of Pediatrics David Geffen School of Medicine at the University of California Los Angeles CA USA; ^2^ Department of Pediatrics, Faculty of Medicine Vajira Hospital Navamindrahiraj University Bangkok Thailand; ^3^ Department of Medicine, Biostatistics and Biomathematics David Geffen School of Medicine at the University of California Los Angeles CA USA

**Keywords:** BONE HISTOLOGY, CHILDREN, DIALYSIS, PRIMARY KIDNEY DISEASE

## Abstract

Congenital diseases of the kidney and urinary tract (CAKUT) and glomerulonephritis are the main causes of chronic kidney disease (CKD) in children. Although renal osteodystrophy (ROD) and indices of mineral metabolism have been characterized in dialyzed children, the impact of primary kidney disease on ROD is unknown. We performed a cross‐sectional study of bone biopsies performed in 189 pediatric dialysis patients aged 12.6 ± 5.4 years. Patients were classified into three groups according to primary kidney disease: CAKUT (*n* = 82), hereditary (*n* = 22), or glomerular disease (*n* = 85). Serum concentrations of calcium, phosphate, alkaline phosphatase (ALP), parathyroid hormone (PTH), and 25(OH) vitamin D were measured at the time of biopsy. Fibroblast growth factor 23 (FGF23) levels were measured in a subset of 59 patients. Levels of calcium, phosphate, PTH, and 25(OH) vitamin D were similar across groups. CAKUT patients had higher serum ALP and lower C‐terminal FGF23 levels. Bone turnover and bone volume parameters did not differ across groups. However, osteoid volume (OV/BV), osteoid surface (OS/BS), and osteoid maturation time (OMT) were highest in the CAKUT group and lowest in the hereditary group. Multiple regression analysis revealed that calcium, phosphate, ALP, and PTH were independently associated with OV/BV and osteoid thickness (O.Th). PTH was an independent factor affecting bone formation rate. The relationship between CKD etiology and bone histomorphometric variables was abrogated after adjustment for biochemical parameters in the multivariable models. Overall, bone histology differed according to CKD etiology in the unadjusted analysis; however, this association could not be confirmed independently of biochemical parameters. Although CAKUT patients had a greater mineralization defect with elevated serum ALP levels, longitudinal studies will be needed to elucidate mediation pathways that might be involved in the complex interplay of CKD‐mineral bone disease (MBD). © 2022 The Authors. *JBMR Plus* published by Wiley Periodicals LLC on behalf of American Society for Bone and Mineral Research.

## 1 Introduction

Chronic kidney disease‐mineral bone disease (CKD‐MBD) is defined as a systemic disorder of mineral and bone metabolism due to CKD, which is manifested by abnormalities in bone and mineral metabolism and/or extraskeletal calcifications. Renal osteodystrophy (ROD) refers to CKD‐related alterations in bone morphometry that are quantifiable only by findings on tetracycline‐labeled iliac crest bone biopsy, the gold standard for the diagnosis of bone diseases. A position statement by Kidney Disease International Global Outcomes (KDIGO) in 2006 recommended the inclusion of turnover, mineralization, and volume (TMV) for the classification of the different subtypes of ROD.^(^ ^1^ ^)^ This methodology has been utilized to define the features of ROD across the spectrum of CKD in pediatric patients.^(^ ^2^ ^)^ Moreover, these bone abnormalities become more relevant with CKD progression regardless of the primary kidney disease.^(^ ^2^, ^3^ ^)^

The pathogenesis of CKD‐MBD involves a complex cascade of maladaptive events that results in bone disease, extraskeletal calcification, and adverse cardiovascular outcomes. Alterations in mineral metabolism, driven by changing kidney function, are important drivers of this condition. However, other factors, including inflammation and anemia, also contribute to skeletal and cardiovascular disease.^(^ ^4^ ^)^ Congenital disorders, including anomalies of the kidney and urinary tract (CAKUT), are the most common causes of CKD in children,^(^ ^5^, ^6^ ^)^ but the prevalence of glomerulopathies—which are associated with inflammation and with a faster progression to end‐stage renal disease (ESRD)^(^ ^7^, ^8^ ^)^—increases with age. Although the features of ROD have been well characterized across the spectrum of CKD in children,^(^ ^2^, ^3^ ^)^ the impact of the most common primary kidney diseases on the specific features of pediatric ROD is unknown, except for patients with specific genetic disorders such as cystinosis and autosomal dominant polycystic kidney disease (ADPKD).^(^ ^9^, ^10^, ^11^, ^12^, ^13^ ^)^ Thus, the present cross‐sectional study was designed to assess the potential effects of the underlying primary kidney disease on the different subtypes of ROD in pediatric patients treated with maintenance dialysis.

## 2 Materials and Methods

With approval by the UCLA Human Subject Protection Committee, we conducted a cross‐sectional study that included initial bone biopsies performed at UCLA in pediatric patients treated with dialysis between 1983 and 2014. The biopsies were performed for different research protocols, and informed consents were obtained from all patient/parents. A total of 189 pediatric dialysis patients were included in the study; the features of ROD were previously reported in 161 of these patients.^(^ ^2^ ^)^ Exclusion criteria included treatment with growth hormone or immunosuppressive agents within 6 months of bone biopsy, presence of aluminum staining on bone biopsy, nephrotic range proteinuria, and/or parathyroidectomy within a year of bone biopsy, as previously described.^(^ ^2^ ^)^ Peritoneal dialysis was the predominant dialytic modality, and subjects received calcium carbonate or sevelamer as enteral phosphate binders and active vitamin D sterols as recommended.^(^ ^14^ ^)^ Patients were classified into three groups according to CKD etiology: (i) CAKUT, comprising a spectrum of congenital malformations of the renal system; (ii) hereditary diseases (Alport's syndrome, medullary cystic kidney disease, and branchiootorenal (BOR) syndrome); and (iii) glomerular diseases, including glomerulonephritis, hemolytic uremic syndrome, and nephrotic syndrome. Patients with cystinosis and ADPKD have specific lesions of ROD^(^ ^9^, ^10^, ^11^, ^12^, ^13^ ^)^ and therefore were excluded from the analysis.

Serum biochemical parameters (calcium, phosphate, alkaline phosphatase [ALP], parathyroid hormone [PTH], and 25(OH) vitamin D) were measured at the time of the biopsy. Calcium, phosphate, and ALP were measured with an Olympus AU5400 chemistry analyzer (Olympus America Inc., Center Valley, PA, USA). PTH levels were measured by the first‐generation Nichols assay, which detects full‐length PTH as well as large amino‐terminally truncated fragments (normal range 10–65 pg/mL).^(^ ^15^, ^16^ ^)^ Results from this first‐generation PTH assay correlate well with those from the second‐generation immunometric PTH assay that specifically detects only the biologically active, full‐length PTH (1‐84), and both assays have similar correlations with bone formation rate.^(^ ^15^ ^)^ C‐terminal (total) FGF23 levels were evaluated in a subset of 59 patients in whom plasma samples—obtained at the time of bone biopsy and stored at −80**°**C—were available. FGF23 concentrations were determined using a C‐terminal FGF23 ELISA kit (Quidel, San Diego, CA, USA), which detects both full‐length, intact FGF23 and C‐terminal FGF23 fragments (reference mean ± standard deviation [SD], 73 ± 38 RU/mL).^(^ ^17^ ^)^

Bone biopsy samples were obtained from the anterior iliac crest biopsy (2 cm below the anterior superior iliac spine) using a modified Bordier trephine needle, and therapy with active vitamin D sterols was discontinued for 4 weeks before the procedure, as previously described.^(^ ^18^ ^)^ Double tetracycline‐labeling was given (10 mg/kg/d for 2 days, followed by a 10‐day tetracycline‐free period, then a subsequent relabeling with 10 mg/kg/d for 2 days; the biopsy was performed 2 to 4 days after the last dose). Bone samples were 0.5 cm in diameter and 1 to 2 cm in length. Specimens were dehydrated in alcohol, cleared with xylene, and embedded in methylmethacrylate. All biopsies were evaluated by the same individual (RP) in undecalcified 5 μm sections treated with Masson‐Goldner trichome stain or toluidine blue; tetracycline labeling was assessed in unstained 10 μm sections. Primary bone histomorphometric parameters were assessed in trabecular bone under 200× magnification using the OsteoMetrics system (OsteoMetrics, Decatur, GA, USA). We used the TMV classification to define each of the different subtypes of ROD as previously reported.^(^ ^2^ ^)^ Bone turnover was determined by bone formation rate/bone surface after tetracycline labeling. Mineralized bone was defined by green‐staining areas; red‐staining seams at least 1.5 μm wide were included for measurement of unmineralized osteoid. Normal values for all histomorphometric parameters were previously obtained from 31 pediatric patients with normal kidney function who underwent elective orthopedic surgery with double tetracycline labeling before bone biopsy.^(^ ^19^ ^)^

### 2.1 Statistical analyses

Normally distributed variables are presented as means ± SD, whereas non‐normally distributed variables are presented as medians with interquartile range (IQR). Bone biochemical parameters and bone histomorphometry were compared among the three groups of primary kidney diseases by the Kruskal‐Wallis test. Spearman correlation coefficients were used to evaluate the correlations between bone biochemical data and bone histomorphometry. A simple linear regression analysis was done to identify potentially significant predictors affecting bone homeostasis. To determine the predictive significance of primary kidney diseases on bone histological variables—while adjusting for other biochemical parameters, time on dialysis, and age—multiple regression analysis was performed. Multicollinearity was formally evaluated using the variance inflation factor (VIF). All VIF values were close to 1, confirming that there was no multicollinearity among the variables. Final models were selected using backward variable selection and *p* < 0.15 as the retention criterion. Log‐transformation was applied to all non‐normally distributed variables before analysis. All statistical analyses were performed using SAS 9.4 (SAS Institute Inc., Cary, NC, USA), and a two‐sided alpha cut‐off of 0.05 was used to determine statistical significance for all tests.

## 3 Results

### 3.1 Demographics

Patient characteristics are depicted in Table 1. The cohort included 189 pediatric dialysis patients, with a mean ± SD age of 12.6 ± 5.4 years; the hereditary group was slightly older than the CAKUT group. Patients were treated with dialysis for a median (IQR) of 16.4 (7.4–44.8) months at the time of bone biopsy; 85% of subjects were treated with peritoneal dialysis; 39% of the cohort was female; and 63% was Hispanic. The distribution of the primary causes of CKD was as follows: CAKUT 43.3%, glomerular diseases 44.9%, and hereditary diseases 11.6%.

**Table 1:** Patient Demographic and Primary Kidney Disease

Parameter	Non‐glomerular		Glomerular
	CAKUT (*n* = 82)	Hereditary (*n* = 22)	(*n* = 85)
Age (years)	11.7 ± 5.2	13.2 ± 5.0^a^	13.1 ± 5.1
Time on dialysis (months)	11.9 (4.6, 41.7)	10.1 (4.3, 34.5)	18.7 (9.0, 34.5)
Sex (female)	26 (32%)	8 (36%)	39 (46%)
Ethnicity			
White	17 (21%)	8 (36%)	18 (21%)
Hispanic	54 (67%)	12 (55%)	55 (65%)
Black	5 (6%)	0 (0%)	9 (11%)
Asian	5 (6%)	2 (9%)	3 (4%)
Primary kidney disease	Renal dysplasia/hypoplasia 35 (43%)	Alport's syndrome 13 (59%)	Nephrotic syndrome 45 (53%)
	Obstructive uropathy 35 (43%)		Glomerulonephritis 32 (38%)
	Reflux nephropathy 10 (12%)	Medullary cystic kidney disease 4 (18%)	Hemolytic uremic syndrome 6 (7%)
	Multicystic dysplastic kidney 2 (2%)	BOR syndrome 5 (23%)	IgA nephropathy 2 (2%)

CAKUT = congenital anomalies of the kidney and urinary tract; BOR = branchiootorenal.

Data presented as means ± standard deviation, median (interquartile range), and numbers and percentages.

^a^ *p*<0.05 vs. CAKUT.

### 3.2 Biochemical parameters

Biochemical parameters are displayed in Table 2. Concentrations of serum calcium, phosphate (age‐adjusted *Z*‐scores),^(20^ ^)^ PTH, and 25(OH) vitamin D did not differ among groups. The CAKUT group had significantly higher serum ALP (age‐ and sex‐adjusted *Z*‐scores)^(^ ^20^ ^)^ and significantly lower plasma FGF23 levels compared with the other two groups.

**Table 2:** Biochemical Parameters According to Primary Kidney Disease

Parameter	Non‐glomerular		Glomerular (*n* = 85)	Kruskal–Wallis *p* value
	CAKUT (*n* = 82)	Hereditary (*n* = 22)		
Calcium (mg/dL)	9.0 ± 0.9 (*n* = 77)	9.3 ± 1.0 (*n* = 21)	9.0 ± 1.1 (*n* = 79)	0.43
Phosphate (age‐adjusted *Z*‐scores)	0.05 ± 0.84 (*n* = 76)	0.30 ± 1.37 (*n* = 20)	0.33 ± 0.99 (*n* = 79)	0.15
Alkaline phosphatase (age‐adjusted *Z*‐scores)	1.63 (−0.29, 5.45) (*n* = 77)	−0.78 (−1.4, 0.7)^a^ (*n* = 20)	0.08 (−1.34, 3.14)^c^ (*n* = 77)	<0.01
Parathyroid hormone (pg/mL)	457 (192, 932) (*n* = 75)	207 (124, 449)^a^ (*n* = 20)	414 (143, 844) (*n* = 75)	0.09
25(OH) vitamin D (ng/mL)	20.3 ± 10.8 (*n* = 44)	16.0 ± 8.9 (*n* = 11)	17.7 ± 11.5 (*n* = 45)	0.27
C‐terminal FGF23 (RU/mL)	64 (34, 270) (*n* = 28)	546 (86, 865)^a^ (*n* = 6)	398 (115, 2270)^c^ (*n* = 25)	<0.01

CAKUT = congenital anomalies of the kidney and urinary tract. Data presented as means ± standard deviation or median (interquartile range).

^a^ *p* < 0.05 for hereditary diseases versus CAKUT.

^b^ *p* < 0.05 for hereditary diseases versus glomerular diseases.

^c^ *p* < 0.05 for glomerular diseases versus CAKUT.

### 3.3 Bone histomorphometric parameters: turnover and volume

As shown in Table 3, bone turnover was elevated in all groups, and bone volume was within the normal range. Values did not differ among the groups.

**Table 3:** Bone Histomorphometric Variables According to Primary Kidney Disease

Parameter	Non‐glomerular		Glomerular (*n* = 85)	Kruskal–Wallis *p* value	Normal range
	CAKUT (*n* = 82)	Hereditary (*n* = 22)			
Bone turnover					
BFR/BS (μm^3^/μm^2^/yr)	79.5 (31.7, 124.4)	51.1 (25.5, 81.0)	59.9 (16.0, 94.5)	0.18	8.0–73.4
Bone mineralization					
OV/BV (%)	8.6 (5.0, 14.0)	4.9 (3.2, 6.7)^a^	6.9 (3.9, 11.2)^b^	0.01	0.2–5.9
OS/BS (%)	47.7 ± 18.0	32.9 ± 11.1^a^	43.5 ± 18.4^b^	<0.01	4.3–31.7
O.Th (μm)	13.4 (9.7, 20.4)	10.2 (8.3, 12.5)^a^	11.4 (9.2, 14.8)^c^	0.01	2.0–13.2
OMT (d)	13.6 (9.3, 23.6)	10.1 (6.4, 12.5)^a^	11.2 (8.2, 15.1)^c^	<0.01	1.2‐11.5
MLT (d)	39.8 (23.2, 82.7)	27.8 (14.5, 44.3)^a^	31.5 (20.3, 78.7)	0.17	2.3–63.8
Bone volume					
BV/TV (%)	28.9 (25.3, 35.5)	27.4 (20.6, 32.5)	26.4 (22.0, 34.7)	0.07	8.9–34.4
Tb.Th (μm)	150 ± 32	139 ± 25	142 ± 32	0.08	72–177
Tb.n (/mm)	2.0 (1.7, 2.2)	1.9 (1.7, 2.1)	1.9 (1.7, 2.2)	0.83	1.3–2.7
Tb.Sp (μm)	351 (288, 429)	366 (317, 477)	367 (301, 455)	0.42	299–587
Bone fibrosis					
Fb/TV (%)	1.00 (0.00, 1.00)	0.00 (0.00, 1.00)	0.15 (0.00, 1.00)	0.27	0

CAKUT = congenital anomalies of the kidney and urinary tract; BFR/BS = bone formation rate/bone surface; OV/BV = osteoid volume/bone volume; OS/BS = osteoid surface/bone surface; O.Th = osteoid thickness; OMT = osteoid maturation time; MLT = mineralization lag time; Tb.Th = trabecular thickness; Tb.n = trabecular number; Tb.Sp = trabecular space; Fb/TV = fibrosis/total volume.

Data presented as means ± standard deviation or median (interquartile range).

^a^ *p* < 0.05 for hereditary diseases versus CAKUT.

^b^ *p* < 0.05 for hereditary diseases versus glomerular diseases.

^c^ *p* < 0.05 for glomerular diseases versus CAKUT.

### 3.4 Bone histomorphometric parameters: mineralization

Impaired mineralization was defined as an abnormality in static indices (osteoid volume/bone volume [OV/BV]) in combination with impaired rates of osteoid mineralization (osteoid maturation time [OMT] or mineralization lag time [MLT]), as previously reported.^(^ ^17^ ^)^ Overall, indices of abnormal mineralization were present in all groups; however, the CAKUT group had significantly higher parameters of unmineralized bone (Table 3). Additionally, the glomerular group had significantly higher static osteoid indices than the hereditary diseases group.

### 3.5 Correlation coefficients of bone histomorphometric variables

Correlation coefficients of bone histomorphometric variables are displayed in Table 4. Age at initiation of dialysis was not associated with bone turnover but was inversely correlated with osteoid volume (OV/BV). Serum calcium levels were inversely correlated with bone formation. However, serum phosphate, ALP (*Z*‐scores), and PTH were positively associated with bone formation. Serum calcium levels were inversely correlated with OV/BV and osteoid thickness (O.Th). Moreover, ALP was positively correlated with OV/BV, O.Th, and OMT but was not correlated with MLT. PTH was also directly associated with all mineralization parameters. Conversely, FGF23 levels were inversely correlated with OV/BV, O.Th, OMT, and MLT.

**Table 4:** Spearman Correlation Coefficients for Bone Histomorphometric Parameters

Parameter	BFR/BS	OV/BV	O.Th	OMT	MLT
Age (years)	0.00	**−0.17** ^a^	−0.06	0.02	−0.14
Calcium (mg/dL)	**−0.23** ^a^	**−0.34** ^a^	**−0.34** ^a^	−0.14	0.04
Phosphate (*Z*‐scores)	**0.22** ^a^	0.02	0.07	−0.08	**−0.23** ^a^
Alkaline phosphatase (*Z*‐scores)	**0.46** ^a^	**0.48** ^a^	**0.49** ^a^	**0.21** ^a^	−0.06
Parathyroid hormone (pg/mL)	**0.62** ^a^	**0.50** ^a^	**0.55** ^a^	**0.19** ^a^	**−0.21** ^a^
C‐terminal FGF23 (RU/mL)	−0.24	**−0.55** ^a^	**−0.60** ^a^	**−0.58** ^a^	**−0.33** ^a^

BFR/BS = bone formation rate/bone surface; OV/BV = osteoid volume/bone volume; OS/BS = osteoid surface/bone surface; O.Th = osteoid thickness; OMT = osteoid maturation time; MLT = mineralization lag time.

^a^ *p* < 0.05 for the Spearman correlation coefficients.

### 3.6 Multivariable regression analysis of determinants of bone turnover, bone mineralization, and bone volume

#### 3.6.1 Determinants of bone turnover

By multiple regression analysis, PTH was positively associated with bone formation rate per bone surface (BFR/BS) (*R* ^2^ = 0.60) (Table 5).

**Table 5:** Multivariable Regression Analysis of Determinants of Bone Formation Rate Per Bone Surface (BFR/BS), Osteoid Volume Per Bone Volume (OV/BV), and Osteoid Thickness (O.Th)

	Prediction of log BFR/BS (*R* ^2^ = 0.38)	Prediction of log OV/BV (*R* ^2^ = 0.51)	Prediction of log O.Th (*R* ^2^ = 0.48)
Predictor (per SD)	% change (95% CI)	% change (95% CI)	% change (95% CI)
CKD etiology: glomerular versus CAKUT	−4.3 (−32.9, 36.5) *p* = 0.81	10.2 (−10.9, 36.3) *p* = 0.37	−5.9 (−15.9, 5.2) *p* = 0.28
CKD etiology: hereditary versus CAKUT	55.9 (−9.5, 168.4) *p* = 0.11	−0.8 (−28.0, 36.8) *p* = 0.96	−7.1 (−21.5, 9.9) *p* = 0.39
Calcium	n/a	−19.4 (−27.2, −10.8) ***p* < 0.001**	−7.8 (−12.6, −2.8) ***p* = 0.003**
Phosphate *Z*‐scores	n/a	−9.4.9 (−18.7, 0.9) *p* = 0.074	−4.0 (−9.2, 1.6) *p* = 0.16
Alkaline phosphatase *Z*‐scores	13.9 (−5.0, 36.7) *p* = 0.16	20.7 (8.2, 34.6) ***p* = 0.001**	6.4 (0.5, 12.7) ***p* = 0.035**
Log parathyroid hormone	113.2 (77.7, 155.8) ***p* < 0.001**	46.0 (30.2, 63.7) ***p* < 0.001**	24.7 (17.5, 32.3) ***p* < 0.001**
Age	n/a	−12.8 (−21.4, −3.3) ***p* = 0.011**	n/a
Log months on dialysis	n/a	−13.7 (−22.1, −4.4) ***p* = 0.006**	−8.1 (−12.7, −3.3) ***p* = 0.002**

CI = confidence interval; CKD = chronic kidney disease; CAKUT = congenital anomalies of the kidney and urinary tract; n/a = not applicable (indicates the variable was not significant upon backward selection and was dropped from the model).

Multicollinearity was assessed using the variance inflation factor. There was no evidence of multicollinearity among the variables.

#### 3.6.2 Determinants of bone mineralization

Lower serum calcium, lower serum phosphate, higher log ALP, higher log PTH levels, and less time on dialysis were all significantly associated with both higher log OV/BV (*R* ^2^ = 0.71) and higher log O.Th (*R* ^2^ = 0.69). CKD etiology, however, was not associated with BFR/BS, OV/BV, or O.Th after controlling for biochemical covariables, age, and time on dialysis.

## 4 Discussion

ROD is a universal complication of CKD in children, contributing to increased fracture rates, bone deformities, bone pain, growth failure, and cardiovascular disease.^(^ ^21^, ^22^ ^)^ Although a few orphan inherited renal diseases have been shown to induce specific mineral and bone abnormalities,^(^ ^9^, ^23^ ^)^ little is known about the precise impact of the most common causes of CKD on the skeleton.^(^ ^24^ ^)^

Primary kidney diseases were classified into three groups: CAKUT, hereditary diseases, and glomerulopathies. CAKUT and glomerular diseases were the most common causes of ESRD, as previously reported.^(^ ^5^, ^6^ ^)^ In the present cohort, CAKUT patients were younger, but dialysis vintage did not differ between groups. Overall, biochemical parameters were similar among groups; however, the CAKUT group had higher ALP and lower total FGF23 levels. Among the hereditary group, we have excluded patients with cystinosis and ADPKD because they have specific alterations of bone and mineral metabolism previously described.^(^ ^9^, ^10^, ^11^, ^12^, ^13^ ^)^

Bone formation rates were similarly elevated in all groups; indices of unmineralized bone were elevated also, but the highest values were observed in the CAKUT group; and bone volume was within the normal range in all groups. Multiple regression analyses demonstrated that serum levels of calcium, phosphate, ALP, and PTH, as well as time on dialysis, were independent predictors of unmineralized bone, whereas PTH was also an independent predictor of bone turnover. However, after controlling for biochemical variables, bone histomorphometric parameters of turnover, mineralization, and volume were not directly affected by CKD etiology. Given that ALP was associated with both CAKUT and indices of abnormal bone mineralization, it may be hypothesized that ALP mediated the observed effect of CAKUT on bone mineralization. For this mediation pathway, serum alkaline phosphate levels may act as a specific marker of the mineralization defect in CAKUT patients. Because mediation is a process that unfolds over time, there have been strong recommendations for the use of longitudinal mediation models that capture the temporal sequence of events.^(^ ^25^ ^)^ Thus, future longitudinal studies will be needed to formally evaluate possible mediation effects in an unbiased manner. Interestingly, time on dialysis was associated with improved indices of mineralization, possibly representing the effects of different ROD therapies.

As FGF23 levels were determined in only a subset of patients, these values were not included in the multivariable analyses. The highest FGF23 levels were observed in the glomerular group, as previously reported,^(^ ^26^, ^27^ ^)^ and the lowest FGF23 levels were observed in the CAKUT group. Multiple bone and mineral‐related factors are involved in the regulation of FGF23; however, several non‐mineral factors, such as inflammation, iron status, anemia, and erythropoietin, have been identified as potentially important determinants of FGF23 production.^(^ ^4^ ^)^ Inflammation can stimulate FGF23 synthesis by activating hypoxia‐inducible factor 1α (HIF1α) to increase FGF23 gene transcription.^(^ ^28^, ^29^ ^)^ Another inflammatory pathway involving activation of the nuclear factor kappa‐light‐chain‐enhancer of activated B cells (NF‐kB) has been shown to increase FGF23 transcription in vitro.^(^ ^28^ ^)^ Corticosteroid therapy may also contribute to elevated FGF23 levels; however, none of the patients received corticosteroids therapy for at least 1 year before the bone biopsy.^(^ ^27^ ^)^ These factors may have partially contributed to the higher FGF23 levels observed in the glomerular group.

CAKUT is the most common cause of ESRD in children.^(^ ^5^ ^)^ The primary etiology of CKD may be a determinant of rates and patterns of CKD progression; indeed, progression to ESRD is longer in CAKUT patients.^(^ ^7^, ^30^ ^)^ Because these patients lack the confounding effects of inflammation and/or other immunological abnormalities, the observed skeletal abnormalities may represent the long‐term consequences of progressive nephron loss in patients with a lifelong disease. Although we have previously described^(^ ^17^ ^)^ a high prevalence of mineralization defects in dialysis patients,^(^ ^2^ ^)^ the results of the present study emphasize for the first time that the mineralization defect is more severe in CAKUT patients and characterized by increases in serum alkaline phosphatase and lower FGF23 levels. It is interesting to note that we and others have previously shown that higher FGF23 levels have been associated with preserved indices of mineralization.^(^ ^17^, ^31^ ^)^ Although skeletal mineralization is not currently assessed by any of the available imaging techniques, a combination of serum alkaline phosphatase and FGF23 levels may potentially serve as more specific biomarkers of skeletal defects in CAKUT patients.

Although fracture rates, skeletal deformities, and growth failure are common in pediatric dialysis patients,^(^ ^21^, ^22^ ^)^ the specific role of the mineralization defect has not been defined. Importantly, such lesions persist despite adequate control of secondary hyperparathyroidism^(^ ^32^ ^)^ and after successful renal transplantation.^(^ ^33^ ^)^ Bone deformities, growth defects, and fractures have also been observed in patients with hypophosphatemic rickets (XLH) and normal renal function. Excess osteoid accumulation develops early in the course of CKD, despite normal levels of calcium, phosphate, PTH, bicarbonate, and vitamin D; however, several osteocytic proteins that regulate phosphate, vitamin D metabolism, and bone turnover are upregulated in addition to elevated circulating FGF23 levels.^(^ ^3^ ^)^ The pathogenesis of this process remains to be defined; indeed, there is a complex interplay between osteocytic proteins such as FGF23, dentin matrix protein 1 (DMP1), and matrix extracellular phosphoglycoprotein (MEPE), among others, that may directly or indirectly affect the mineralization process in CKD.

In summary, we have demonstrated that, after controlling for biochemical covariables, bone histomorphometric parameters of turnover, mineralization, and volume were not affected by CKD etiology in pediatric dialysis patients. However, we have shown that the mineralization defect is more severe in CAKUT patients and is characterized by elevated serum alkaline phosphatase and lower FGF23 levels. The relationship between CAKUT and the bone mineralization defect may be mediated through alkaline phosphatase. However, mediation analysis performed in longitudinal studies is needed to better define this relationship and its potential clinical implications.

## Disclosures

None of the authors have conflict of interest.

5

### Peer Review

The peer review history for this article is available at https://publons.com/publon/10.1002/jbm4.10601.
